# Clinical Audit of Manipulation of Paediatric Forearm Fractures in the Emergency Department at a District General Hospital: A Quality Improvement Project

**DOI:** 10.7759/cureus.51095

**Published:** 2023-12-25

**Authors:** Omer Nasim, Roanna Norman, Rohit Gangadharan, Thet Paing Oo, Joanna Kalderon, Abdallah Alhamarsheh, Ashwin John, Nichola Kelsall

**Affiliations:** 1 Trauma and Orthopaedics, University Hospital Southampton NHS Foundation Trust, Southampton, GBR; 2 Trauma and Orthopaedics, Poole Hospital, University Hospitals Dorset NHS Foundation Trust, Poole, GBR

**Keywords:** paediatric fractures, manipulation of fractures, quality improvement projects, distal forearm pediatric fracture, paediatric forearm fractures

## Abstract

Background

Early reduction of paediatric forearm fractures under procedural analgesia has the benefit of avoiding admission and general anaesthesia. In addition to lowering the risks of treatment and reducing the number of treatment episodes, this approach also reduces the psychological stresses on the child and the parents. British Orthopaedic Association Standards for Trauma and Orthopaedics (BOAST) and Getting It Right First Time (GIRFT) guidelines recommend that all units managing paediatric fractures should have protocols to facilitate procedural analgesia for manipulation of forearm fractures. A recent standard operating procedure has been created for this purpose and has outlined local standards to adhere to. Regular audits of paediatric manipulations in the emergency department must be undertaken in line with GIRFT recommendations. The aim is to identify potential barriers to implementation, which can be improved, and to ensure that a high standard of care is delivered.

Aim

The aim of this study was to assess the effects of the introduction of local guidelines on the manipulation of paediatric fractures in the emergency department, to assess the adherence of the emergency/orthopaedic departments with these guidelines, and to assess the outcome of all childhood forearm manipulations at University Hospitals Dorset (UHD), to help guide further practice.

Material and methods

This was a retrospective and prospective study in which the patients admitted to Poole Hospital, Poole, United Kingdom were identified according to the criteria and were analyzed in three separate groups in terms of pre-implementation and post-implementation. Patients were gathered from the orthopaedic on-call trauma lists. All paediatric patients who had a forearm fracture were included (including those who were not manipulated).

The first group was the surveillance group in which a clinical audit was completed to review if any of the paediatric patients with forearm fractures were being manipulated in accident and emergency (A&E). The second group included the patients for whom the first standard operating procedure documentation was initiated with the intention of improving the service provided and reduce the number of paediatric forearm fractures going to theatre for simple manipulation and prevent a general anaesthetic. The third group was to review the established pathway and to see which areas of the pathway needed focus to make it better and more in line with the flow of patients through the emergency department. These plan, do, study, act (PDSA) cycles took place from March 2022 to March 2023. Paediatric patients with open or neurovascular damage were excluded from the cohort.

The findings and the data were analysed in Microsoft Excel (Microsoft Corporation, Redmond, Washington, United States) and presented through regional meetings to discuss the progress and potential changes in making the pathway by involving all the stakeholders, i.e., the emergency department, orthopaedic department, and theatre managers.

Results

An overall reduction was seen in paediatric forearm fractures going to theatre. Almost 30% of the forearm fractures were attended to in the emergency department, identification of factors that affect the numbers was quantified, and improvement in documentation throughout the PDSA cycles was observed.

## Introduction

Pediatric bone injuries are a common occurrence in the emergency department (ED), with over 30% of children experiencing at least one fracture before reaching the age of 17 years [1]. Among these fractures, forearm fractures are particularly prevalent in children, accounting for nearly 30% of cases [1-3]. Notably, distal fractures of the metaphyseal radius are the most frequently encountered type. Several studies have demonstrated the effectiveness of managing these fractures in the ED [4,5], showcasing successful reduction procedures that result in reduced hospitalizations and decreased reliance on general anaesthesia.

In the pediatric ED, distal forearm fractures stand out as one of the most commonly seen orthopaedic injuries [6-8]. The primary approach to managing displaced or angulated fractures involves immediate closed reduction and cast immobilization with procedural sedation [4-7]. Numerous long-term follow-up studies have consistently demonstrated that the majority of these fractures can be successfully treated through closed reduction, resulting in healing without complications or long-term issues [9-11].

Recent literature on distal forearm fractures has predominantly focused on outcomes, specifically comparing types of immobilizations such as removable splints or plaster immobilization for compression fractures and evaluating different cast lengths for reduced fractures (below-elbow or above-elbow) [3]. Published studies indicate a considerable range (7-39%) in the rates of secondary displacement of distal radial fractures, with many managed conservatively [6,12-14].

For diaphyseal fractures of the forearm in children under the age of 15 years, treatment options include both surgical and conservative approaches [12,15,16]. However, the preferred initial treatment for this type of fracture is conservative, especially considering the greater potential for growth and subsequent remodelling in pediatric patients. Surgical intervention becomes necessary in cases of irreducible fractures, secondary displacement despite splinting, or recurrent fractures [17,18].

In the United Kingdom, a recent decline in the number of junior medical staff, coupled with pressure to meet targets in EDs, has resulted in a majority of children requiring manipulation for fracture reduction being admitted to hospitals. This practice can be distressing for the child and their family, leading to adverse psychological effects and disruptions to daily life. Moreover, extended waiting times for operating theatres, with reported median times exceeding eight hours in some instances, exacerbate the challenges.

However, performing distal forearm fracture reductions in the ED can yield comparable clinical outcomes to hospital admission, provided that reduction is effectively achieved and maintained with a well-moulded plaster cast or splint. This approach not only averts the negative psychological impact on the child and their family but also mitigates the associated high costs linked with hospital admission.

In accordance with the British Orthopaedic Association Standards for Trauma and Orthopaedics (BOASTS) guidelines, units managing children's forearm fractures should establish protocols facilitating early, definitive manipulation and casting, eliminating the need for admission. These protocols must address procedural analgesia and sedation, and ensure a timely response to manipulation. Competent orthopaedic practitioners, as defined by local protocols, should perform forearm fracture manipulations, with a documented case and image review by a consultant orthopaedic surgeon within 48 hours of injury.

While a considerable number of pediatric forearm fractures can be manipulated and cast in the ED during the initial visit, the Getting It Right First Time (GIRFT) report identified significant variation across trusts in the admission and treatment of children aged 16 and under in the operating theatre. The report calculated that England dedicated over 250 weeks of operating time per year to forearm and wrist manipulations between 2016 and 2019, a figure that could potentially be reduced by 80% (to 57 weeks or less) if all trusts adopted well-developed ED manipulation protocols [19].

This standard operating procedure (SOP) serves as a comprehensive guideline for the procedural analgesia-based manipulation of pediatric forearm fractures in the ED. The document meticulously outlines patient selection criteria and procedural details, prioritizing safety and minimizing distress for the child. The associated report underscores the potential for significant efficiencies, estimating an annual cost reduction of £1.6 million for the NHS by decreasing admissions for simple forearm and wrist fractures [19-22].

The SOP emphasizes the advantages of early reduction of paediatric forearm fractures under procedural analgesia, including the avoidance of admission and general anaesthesia. Beyond mitigating treatment risks and reducing treatment episodes, this approach also alleviates psychological stresses for both the child and their parents.

Guidelines from the BOAST and the GIRFT program recommend that all units managing paediatric fractures establish protocols for procedural analgesia during forearm fracture manipulation [20-22]. Consequently, the audit conducted aimed to pinpoint areas of improvement, establishing a pathway to enhance the delivery of procedural sedation and reduce cases requiring theatre intervention in the ED for manipulable forearm fractures.

## Materials and methods

This study was conducted at Poole Hospital, Poole, United Kingdom, within the surgical care group of trauma and orthopaedics. Approval for the audit was obtained from the Department of Clinical Audit at University Hospitals Dorset NHS Foundation Trust in 2022 (Reference #5529). The data collection comprised three cycles: the initial retrospective phase covered trauma lists and database extraction from Symphony software (EMIS Group plc., Leeds, United Kingdom), spanning from July 2022 to August 2022. Subsequent cycles were conducted prospectively after implementing the protocol, developed collaboratively between the ED and the trauma and orthopaedic team. The second and third cycles occurred between September 2022 and March 2023.

A checklist, aligned with GIRFT and BOAST guidelines and informed by a literature review of NHS hospital policies, was utilized for data collection. The study included all forearm fractures in children aged 2-16 years, while exclusion criteria encompassed patients with fractures in acceptable alignment, those requiring complex reduction, cases necessitating surgery, and open fractures demanding debridement (Table 1).

**Table 1:** Patient selection and suitability of fractures for manipulation in the emergency department (ED)

Fractures suitable for manipulation in the ED
Easily reducible fractures
Simple angulated diaphyseal and metaphyseal forearm fractures
Salter-Harris type 1 and 2 fractures
Might be appropriate for manipulation in the ED if definitive treatment is delayed e.g., overnight admissions, no availability of emergency theatres
Improving fracture alignment reduces soft tissue injury and pain and prevents further neurovascular compromise
Fractures not suitable for manipulation in the ED
Fractures that are difficult to reduce
Minimally displaced fractures with remodelling potential
Polytrauma/multiple fractures
Fractures with neurovascular compromise needing surgical exploration
Open fractures often require surgical debridement and fracture fixation in the operation theatre

The procedural analgesia approach employed in this study involved a combination of intranasal (IN) fentanyl and Entonox, which has demonstrated high efficacy in pediatric analgesia for the specified procedures. It was essential for both the child and the caregiver to have a comprehensive understanding of the procedure. Emphasis was placed on ensuring accurate documentation, particularly with regard to obtaining informed written consent and detailing the procedure's execution. This commitment to clarity and documentation ensures a thorough understanding of the process, contributing to the overall safety and effectiveness of the analgesic intervention.

**Figure 1:**
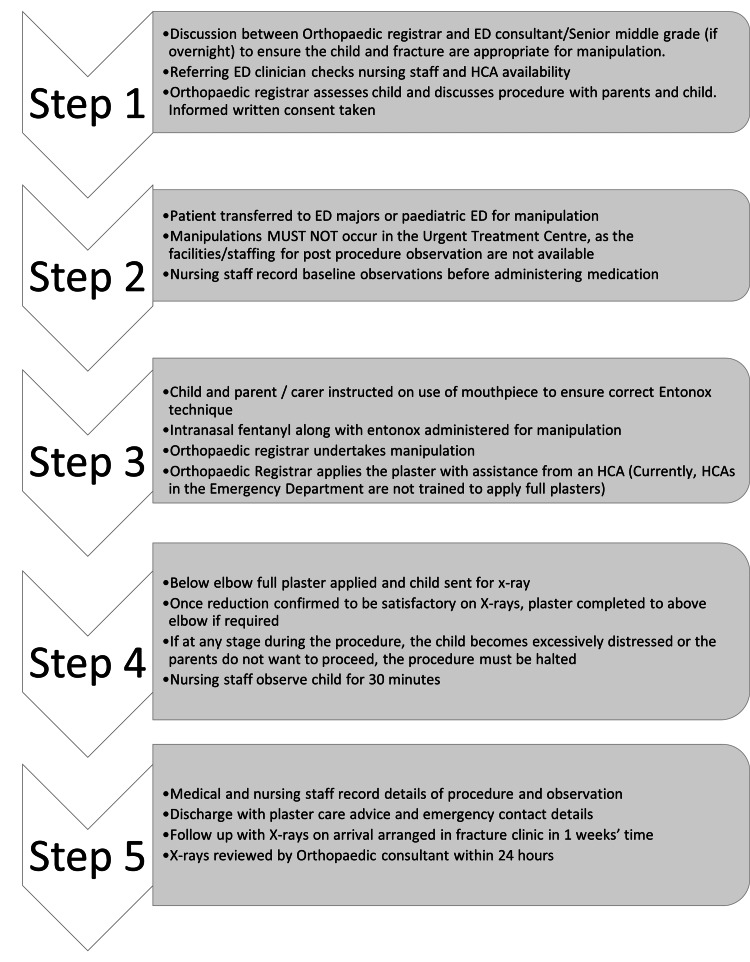
Step-wise approach to the process of going through the paediatric fracture manipulation pathway

Intranasal fentanyl is commonly used at the hospitals of the University Hospitals Dorset NHS Foundation Trust for the application of the plaster cast in the paediatric population. This is quicker to prepare and administer compared to intranasal diamorphine or intravenous morphine, which would require monitoring and is not suitable for children. Therapeutic levels are reached within two minutes and may last up to around 30 minutes, which is adequate for manipulation and plaster cast application. Also, intranasal fentanyl is minimally sedating and short-acting, and a very small dose is used according to the weight of the patient. Entonox is used along with this as it is easy to administer, reaches therapeutic levels quickly, is easily reversible, and stays in the body for less than 30 minutes; thus, cessation of administration is enough to reverse the effects. Figure 1 shows the step-wise paediatric fracture manipulation pathway.

## Results

In the initial analysis round, 11 fractures were identified over two-month periods that were suitable for reduction under procedural sedation (PS) in the ED (Table 2). Subsequent cycles revealed an increased number of patients with fractures owing to perhaps the change in weather (months of outdoor activity) with 11 fractures identified in the second round (Table 3) and nine fractures in the third cycle (Table 4) that could be included in the cohort for reduction for this particular pathway and were manipulable.

**Table 2:** First cycle: establishing the gap and the need for the paediatric pathway implementation

Pre-implementation – 2 months (n=11; 23 excluded due to criteria)			n	%
Gender				
Male			10	90.9%
Female			1	9.1%
Age Range				
0-5 years			2	18.2%
6-10 years			4	36.4%
11-15 years			5	45.5%
Injury				
Both Bone Fracture			8	72.7%
Isolated Radius Fracture			3	27.3%
Manipulation in Emergency Department				
Yes			1	9.1%
No			10	90.9%
Theatre Requirement				
Yes			4	36.4
No			7	63.6

**Table 3:** Second cycle post implementation of the paediatric fracture manipulation pathway

Post-implementation – 3 months (n=74; 139 excluded due to criteria)	n	%
Gender		
Male	52	70.3%
Female	22	29.7%
Age Range		
0-5 years	14	18.9%
6-10 years	38	51.4%
11-15 years	19	25.7%
16-17 years	3	4.1%
Injury		
Both Bone Fracture	48	64.9%
Isolated Radius Fracture	24	32.4%
Isolated Ulna Fracture	2	2.7%
Manipulation in Emergency Department		
Yes	11	14.9%
No	63	85.1%
Theatre Requirement		
Yes	21	28.4%
No	53	71.6%

**Table 4:** Third cycle of the post-implementation of the paediatric fracture pathway to review progress and space for improvement

Post-implementation – 4 months (n=52; 5 excluded due to criteria)	n	%
Gender		
Male	37	71.2%
Female	15	28.8%
Age Range		
0-5 years	6	11.5%
6-10 years	20	38.5%
11-15 years	24	46.2%
16-17 years	2	3.8%
Injury		
Both Bone Fracture	17	32.7%
Isolated Radius Fracture	31	59.6%
Isolated Ulna Fracture	4	7.7%
Manipulation in the Emergency Department		
Yes	9	17.3%
No	-	-
Theatre Requirement		
Yes	1	1.9%
No	-	-

Throughout all cycles, a notable gender disparity was observed, with a higher proportion of males experiencing fractures. The study categorized fractures into age brackets and distinguished between both bone fractures (radius and ulna) and isolated radius fractures.

**Figure 2:**
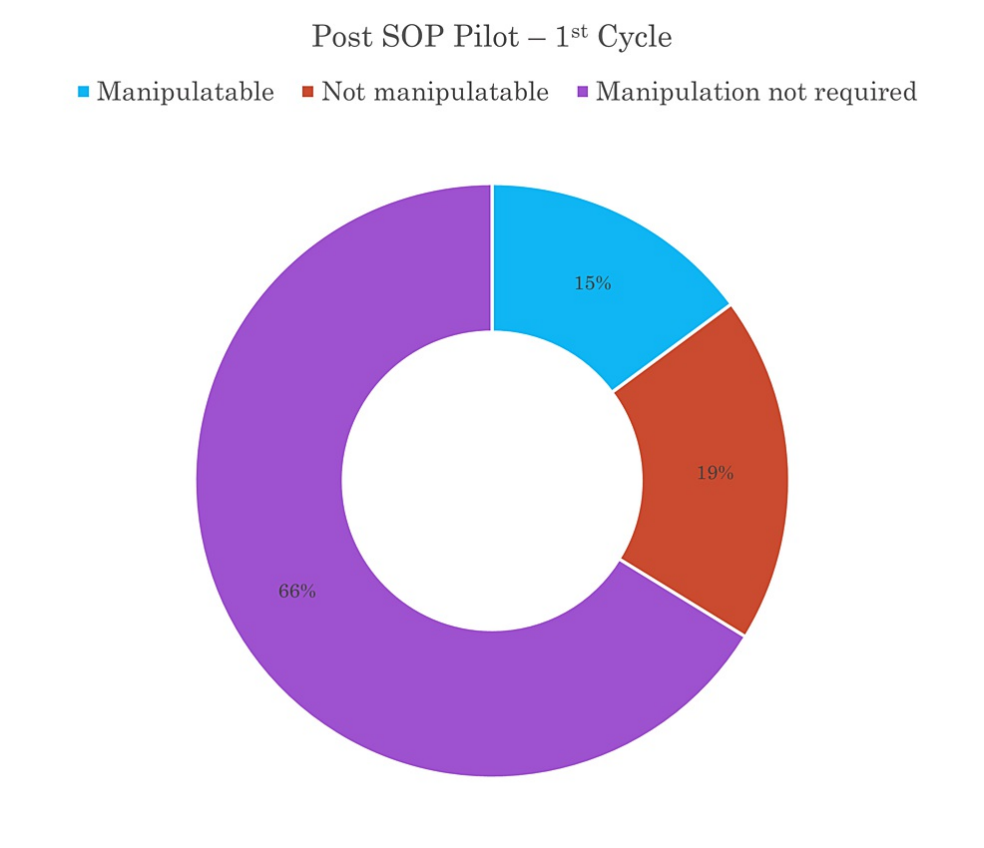
Pie chart showing the percentage of the manipulability of forearm and wrist fractures in the first cycle

**Figure 3:**
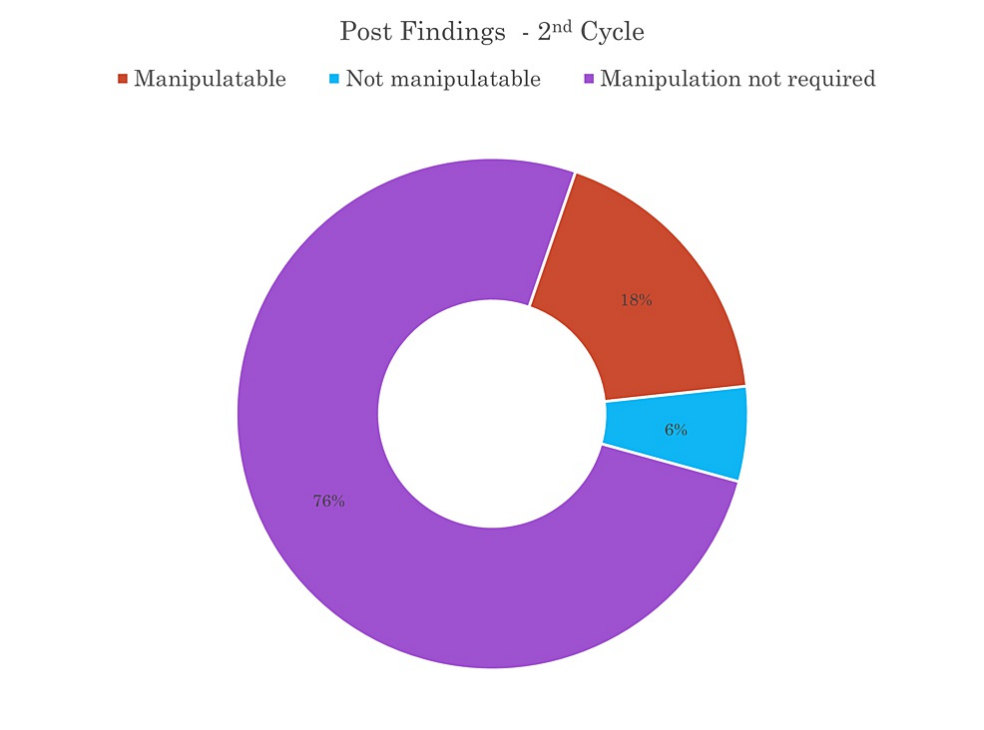
Pie chart showing the percentage of the manipulability of forearm and wrist fractures in the second cycle

**Figure 4:**
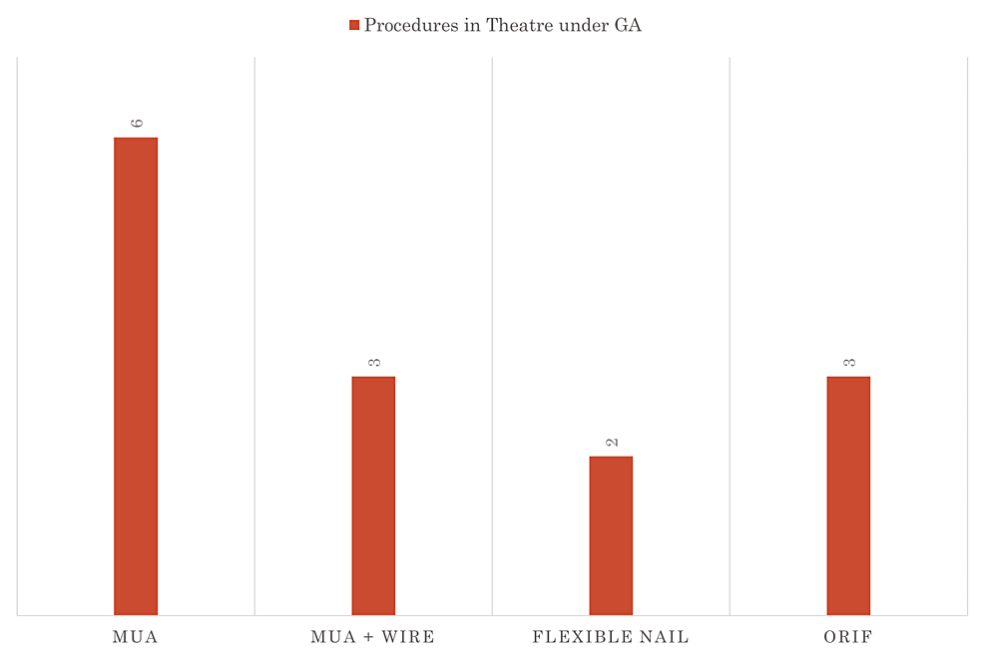
Fractures not manipulatable in the ED and their management ^*^43% of non-manipulatable fractures underwent an MUA in theatre, 21% had an MUA + wiring, and 36% had an ORIF and/or flexible nails. Data given as n MUA: manipulation under anaesthesia; ORIF: open reduction and internal fixation

Radiographs underwent a thorough evaluation by two different senior-level registrars to determine if the fractures could be manipulated under procedural sedation in the ED or if they necessitated manipulation under anaesthesia in the theatre the following day. A quarter of the patients required a decision between whether the patient was in the category of manipulation in ED or would require theatre manipulation (Figure 2). The decision regarding the requirement for theatre intervention was made during the review. Notably, the data across all cycles consistently indicated a reduced need for theatre intervention, with the majority of reviewed fractures deemed amenable to manipulation under procedural sedation in the ED. Figure 3 shows that the number of fractures deemed not amenable to manipulation had decreased and more fractures were being manipulated with the correction procedural sedation. Figure 4 shows that of the fractures that were not manipulated in ED, half were manipulated just under anaesthesia and some required metalwork placement.

**Figure 5:**
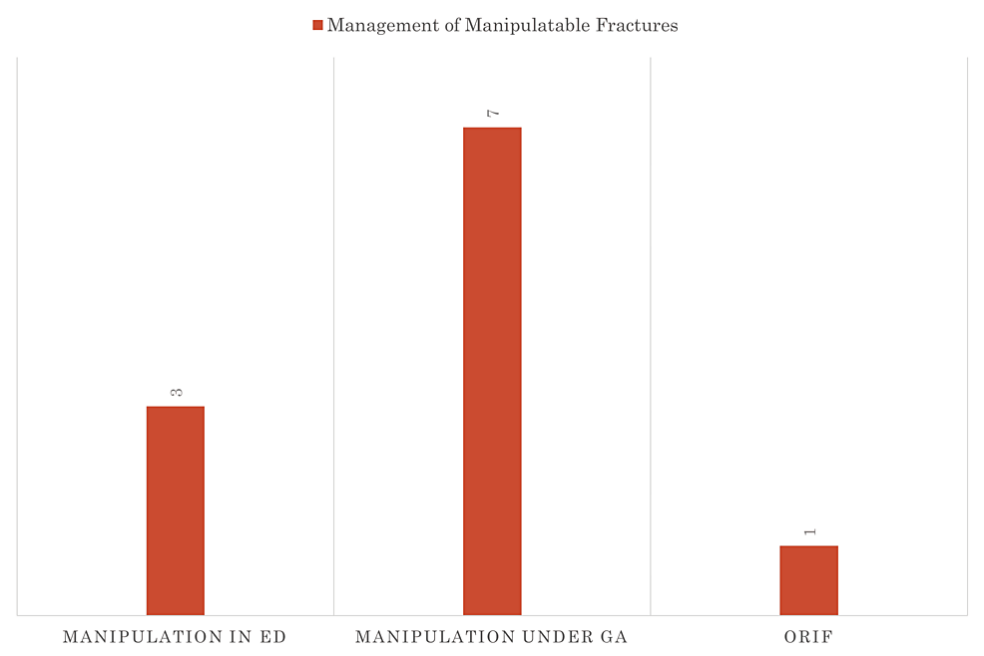
Management of the manipulatable fractures 27% of manipulatable fractures were manipulated in ED. None required theatre after this. *numbers above the bar are the total number of cases (n) ORIF: open reduction and internal fixation; GA: General Anaesthesia

**Figure 6:**
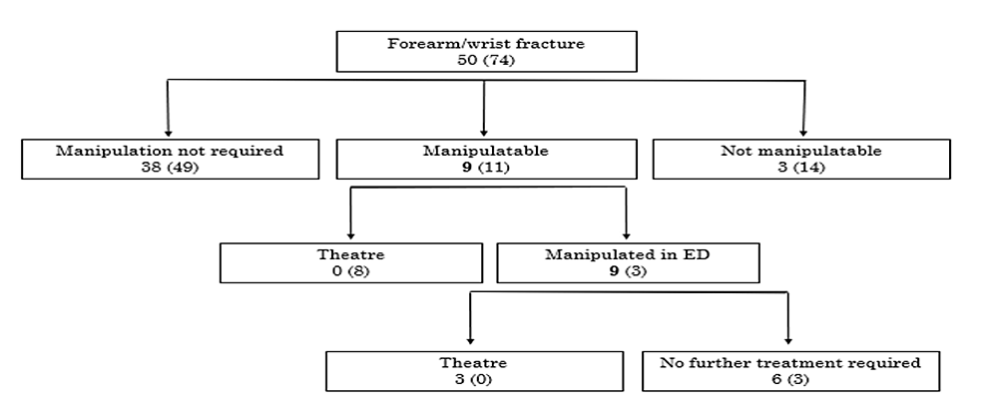
Flowchart representation of the fracture pathway in the second and third cycles Number outside the bracket: third cycle; Number inside the bracket: second cycle *all nine of those manipulable were manipulated in the ED Data has been given as n

The subsequent cycles of the quality improvement initiative revealed key findings indicating a reduced need for theatre interventions, with manipulations increasingly conducted in the emergency department. Notably, all manipulations were performed exclusively by the orthopaedic on-call team. Cases that did not require manipulation were either provided with a plaster cast and discharged for clinic review or were not referred to the on-call team. Figure 5 shows that 27% of manipulatable fractures were manipulated in ED and none of these required theatre after this. Significantly, none of the manipulations were executed by the ED team for the paediatric patient cohort, emphasizing the specialized involvement of the orthopaedic team in this aspect of care. The second and third cycle findings in Figure 6 show that small gains were made in the third cycle with fewer patients requiring theatre post manipulation in ED, which could be postulated as that the teams were getting proficient at using this pathway. 

## Discussion

The audit underscored the necessity for a SOP within the hospital's guidelines. Having an accepted SOP is crucial to prompt stakeholders to follow a universally accepted and established treatment algorithm, as emphasized in various regions [19].

Throughout the study, plan-do-study-act (PDSA) cycles were employed. These cycles involve a four-step method for testing a change, encompassing planning, implementation, observation of results, and acting on the insights gained. This approach aligns with a scientific method for action-oriented learning, facilitating a structured and iterative process for continuous improvement in healthcare practices.

PDSA Cycle 1 involved the initial audit to assess the number of pediatric cases being sent to the theatre for routine manipulations that could feasibly be conducted in an ED setting. Once established, the findings were presented at a local meeting that included key stakeholders such as the emergency department, orthopaedic department, radiology department, and theatre management. Drawing on evidence from other regional centres, it was hypothesized that implementing a standardized pathway would effectively reduce the number of theatre attendances and alleviate the patient load on those services. Subsequently, a provisional pathway was collaboratively developed with consensus from all involved teams and was then tested in the subsequent cycles of the quality improvement project.

PDSA Cycle 2 involved presenting the audit findings after trialling the established pathway. The focus was on demonstrating improvements in the number of completed manipulations and obtaining feedback from both parents and patients who had experienced the pathway. The response was positive, with individuals expressing a preference to avoid admission, theatre attendance, and general anaesthesia.

During this cycle, areas for improvement were identified, particularly in terms of clear documentation of procedures and consent. Ongoing improvements were progressively implemented throughout the patient cohort. Notably, it was observed that parents who received adequate information were more inclined to provide positive feedback, highlighting the importance of clear communication. To address this, a plan was devised to create informational leaflets. Recognizing that absorbing information can be challenging after a traumatic episode, the leaflets were intended to serve as a resource for frequently asked questions post-ED visit, enhancing patient and parent understanding and satisfaction. The step-wise pathway has been tabulated in Table 5. 

**Table 5:** Emergency department distal radius/ulna fracture pathway

Steps	
Step 1	Triage
	Any gross deformity with critical skin or neurovascular compromise? Urgent senior review? Manipulation?
Step 2	Radiographs
	Anteroposterior and Lateral forearm views
Step 3	Criteria Checks
	Suitable forearm fracture for manipulation/meets criteria for procedural sedation/referral to orthopaedic team
Step 4	Discussion/Consent
	Sedation explanation, consenting, preparing the child/guardian for manipulation
Step 5	Procedure
	Sedation involving Entonox/Intranasal Fentanyl prescribed according to guidelines – manipulation & ¾ plaster cast by orthopaedic team and 3-point moulding with 50% overlap of wool. Check for neurovascular status pre-manipulation and clear documentation of all events.
Step 6	Post Procedure
	Post manipulation radiographs and neurovascular assessment and observations to be completed until fit for discharge home – with leaflet and clinic appointment scheduling
Step 7	Follow- up
	Referral to the virtual fracture clinic and subsequent planning To discuss the case with the orthopaedic consultant in the meeting to confirm management plan

Throughout this study, it was established that the combination of intranasal fentanyl and Entonox proved to be a safe and effective treatment for providing analgesia to children aged 4-16 years with distal forearm fractures. This approach facilitated successful manipulation and reduction in the ED. The collaboration between emergency teams and orthopaedic departments contributed to the efficient assessment, treatment, and discharge, aligning with the four-hour target.

**Figure 7:**
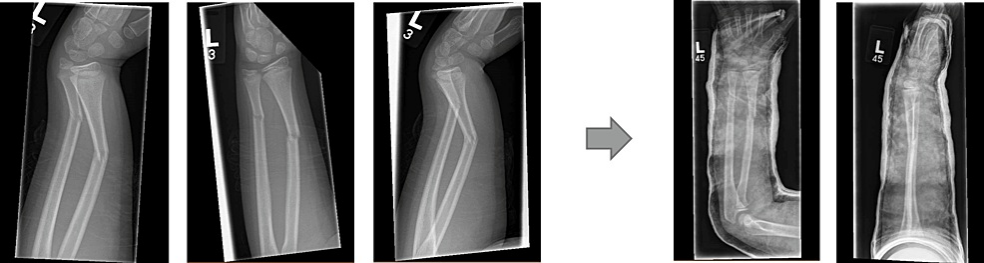
Radiograph 1 showing distal radius/ulna fracture in AP/Lat views and the subsequent manipulated views AP: Anteroposterior; Lat: Lateral

**Figure 8:**
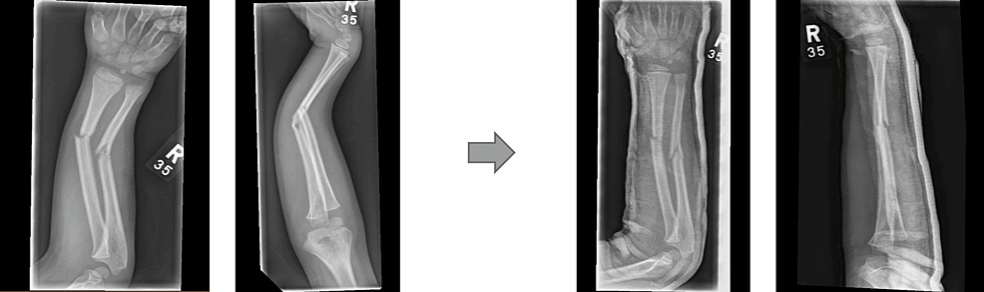
Radiograph 2 showing distal radius/ulna fracture in AP/Lat views and the subsequent manipulated views AP: Anteroposterior; Lat: Lateral

**Figure 9:**
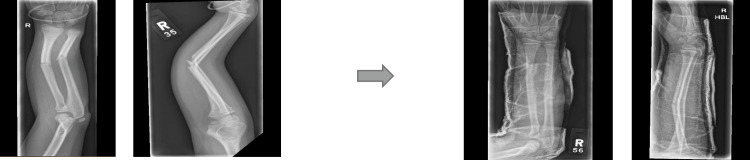
Radiograph 3 showing distal radius/ulna fracture in AP/Lat views and the subsequent manipulated views AP: Anteroposterior; Lat: Lateral

Examples of three different forearm fractures and their subsequent manipulation are shown in Figures 7-9. All of them were completed under the pathway in Table 5 with positive outcomes in terms of patient satisfaction and consultant review in the morning deemed to be managed conservatively and to be followed up in the clinic. 

However, the study has its limitations, including insufficient documentation and the absence of a control group, preventing direct comparisons related to outcomes, compliance, and cost-effectiveness. Based on the findings, a recommendation is made for the implementation of a dedicated plaster room within the ED, staffed by a pediatric nurse. This enhancement could offer substantial benefits to both children and parents by eliminating the need for overnight stays and treatment in the theatre.

## Conclusions

The introduction of a structured pathway for paediatric fractures resulted in a notable 30% decrease in the requirement for theatre interventions. This reduction underscores the pathway's efficacy in minimizing the need for admission and general anesthesia, consequently lowering associated risks and treatment episodes. Aligned with BOAST and GIRFT guidelines, the pathway not only enhances clinical efficiency but also addresses the psychological stress experienced by young patients and their parents. Regular audits further ensure ongoing adherence to guidelines, supporting a commitment to sustained high standards of care. The success of this structured approach signifies a comprehensive and patient-centered strategy in managing paediatric fractures.
